# Alveolar Rhabdomyosarcoma in a 69-Year-Old Woman Receiving Glucagon-Like Peptide-2 Therapy

**DOI:** 10.1155/2015/107479

**Published:** 2015-07-22

**Authors:** Laura E. Zyczynski, Jonathan B. McHugh, Thomas E. Gribbin, Scott M. Schuetze

**Affiliations:** ^1^Department of Internal Medicine, University of Michigan Health System, 1500 E. Medical Center Drive, Ann Arbor, MI 48109, USA; ^2^Department of Pathology, University of Michigan Health System, 1500 E. Medical Center Drive, Ann Arbor, MI 48109, USA; ^3^Mercy Health Lacks Cancer Center, 250 Cherry Street SE, Grand Rapids, MI 489503, USA

## Abstract

A 69-year-old woman was diagnosed with alveolar rhabdomyosarcoma (ARMS) of the nasopharynx. She has a history of catastrophic thromboembolic event in the abdomen that caused short-gut syndrome and dependence on total parenteral nutrition (TPN) twelve hours per day. She was treated for short-gut syndrome with teduglutide, a glucagon-like peptide-2 (GLP-2) analog, which led to reduction of TPN requirements. However, a few months later, she developed metastatic alveolar rhabdomyosarcoma. Though a causative relationship is unlikely between the peptide and ARMS due to the brief time course between teduglutide therapy and sarcoma diagnosis, neoplastic growth may have been accelerated by the GLP-2 analog, causing release of IGF-1. The transmembrane receptor for IGF-1 is frequently overexpressed in ARMS and is implicated in cell proliferation and metastatic behavior. This case describes a rare incidence of metastatic alveolar rhabdomyosarcoma in a sexagenarian and possibly the first case reported associated with the use of teduglutide. Teduglutide was discontinued due to a potential theoretical risk of acceleration of sarcoma growth, and the patient's rhabdomyosarcoma is in remission following sarcoma chemotherapy.

## 1. Introduction

Soft tissue sarcomas are a heterogeneous group of mesenchymal neoplasms that affect patients of all ages. The incidence of soft tissue sarcoma is about 30 per million persons per year; in the United States about 12,000 people are diagnosed with soft tissue sarcoma each year [1]. Rhabdomyosarcoma (RMS) is the most common malignant soft tissue tumor of childhood, comprising approximately 50% of all pediatric soft tissue sarcomas, or about 5% of childhood cancers, but is rare in adults occurring in fewer than 5% of soft tissue sarcoma cases [2, 3]. Males are more likely than females to develop RMS. Rhabdomyosarcoma is most often sporadic, but certain cases are associated with familial syndromes, including Li-Fraumeni syndrome, neurofibromatosis type I, and hereditary retinoblastoma [4]. The two major histologic subtypes of RMS in children are embryonal RMS (ERMS) and alveolar RMS (ARMS). Embryonal RMS has a bimodal age distribution, with more than half of the cases arising before the age of 5 years and a smaller peak in adolescence, whereas ARMS is more likely to occur in adolescence [2].

Treatment of rhabdomyosarcoma is based on risk stratification at the time of diagnosis, which is based on several criteria developed by the Soft Tissue Sarcoma Committee (previously the Intergroup Rhabdomyosarcoma Study Group (IRSG)) of the Children's Oncology Group (COG). Treatment is multidisciplinary in nature, including surgery, radiation therapy, and chemotherapy. Favorable prognostic indicators include gross complete surgical removal of the tumor at the time of diagnosis, tumor size <5 cm, and age older than 1 but less than 10 years at diagnosis. Regional lymph node involvement in patients with alveolar rhabdomyosarcoma and distant metastasis is associated with a less favorable prognosis compared with those patients with localized disease [3].

In adults, soft tissue sarcomas comprise about 1% of solid tumor malignancies, and RMS makes up about only 3% of adult soft tissue sarcomas. If a patient over the age of 18 years is diagnosed with rhabdomyosarcoma, it is much more likely to be pleomorphic (adult-type) RMS than one of the pediatric iterations of the disease. There are case reports in the medical literature about adults with ARMS, but cases are exceedingly rare. Adults with ARMS have a very high risk of developing metastases and usually die from the disease [5]. This report details a case report of an adult woman with alveolar rhabdomyosarcoma of the nasopharynx and discusses the possible role of teduglutide in growth of the sarcoma.

## 2. Clinical Presentation

The patient is a 69-year-old woman who presented with a chief complaint of unilateral clear rhinorrhea for two weeks. She was treated with steroid nasal spray without benefit. Due to persistent symptoms, she was treated with antibiotics for acute sinusitis, again without improvement. A few weeks later, she noted left-sided nasal occlusion with difficulty breathing through her nose and an enlarging left neck mass. She underwent computed tomography scanning of the neck that showed a left nasopharyngeal mass measuring 3 × 2.4 × 2.6 cm, extending from the skull base along the anterior inferior aspect of the left clivus inferiorly through the level of C1-C2. The mass encased the left upper internal carotid artery. A second mass was in the midline on the posterior choana abutting the nasal septum, measuring 2.2 × 2.2 × 1.8 cm, and a mass-like area between the nasal septum and the left middle turbinate was present, measuring 1.7 × 1.6 × 0.5 cm. Left submandibular and upper deep cervical chain adenopathy and a soft tissue mass in the flank detected on fluorodeoxyglucose-positron-emission tomography (FDG-PET) suggested metastatic cancer.

**Figure 1:**
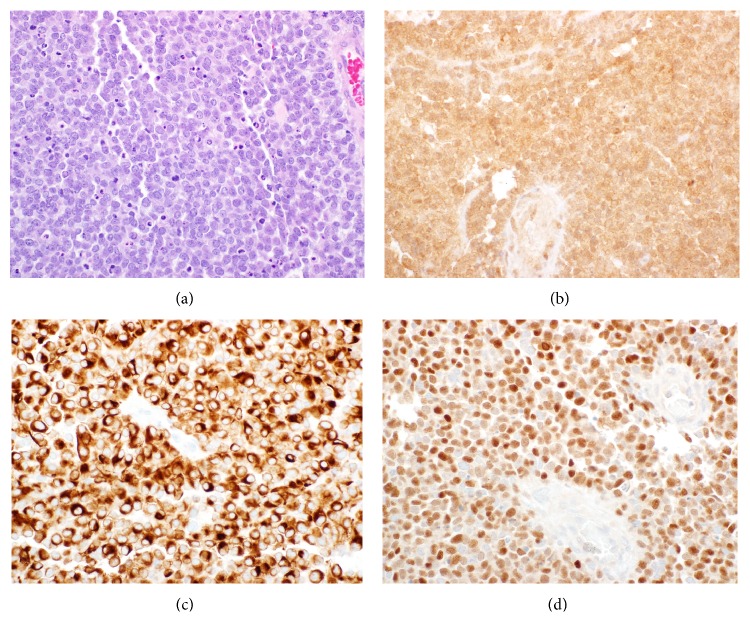
Photomicrographs of diagnostic biopsy demonstrating rhabdomyosarcoma. (a) 40x magnification of hematoxylin- and eosin-stained section demonstrating diffuse small round cells with a high nuclear to cytoplasmic ratio. (b) 40x magnification of horseradish peroxidase immunohistochemical stain for IGF1-R demonstrating uniform expression. (c) 40x magnification of horseradish peroxidase immunohistochemical stain for desmin demonstrating cytoplasmic expression. (d) 40x magnification of horseradish peroxidase immunohistochemical stain for myogenin demonstrating nuclear localization.

An excisional biopsy of the nasopharyngeal mass was performed. Pathology was consistent with ARMS. Immunohistochemical staining was notable for diffuse and strong positivity for CD56, vimentin, desmin, and myogenin (see Figure 1). There was patchy positivity for S-100. Tumor was negative for melan-A, HMB45, and cytokeratin cocktail. An antibody to the insulin-like growth factor-1 receptor reacted with tumor cells. The patient had a fine needle aspiration biopsy performed on the left neck and left flank masses, both of which were positive for metastatic ARMS. A portion of the nasopharyngeal mass biopsy was sent for reverse transcriptase-polymerase chain reaction (RT-PCR) for PAX3/FOX01 translocation chimeric transcript, and this was present, confirming the diagnosis.

The patient has a past medical history of a catastrophic thromboembolic event in the abdomen that resulted in necrotic bowel. She had a partial small bowel resection and subtotal colectomy with placement of ileostomy about 15 months prior to presentation to our medical center. This resulted in short bowel syndrome (SBS) and dependence on total parenteral nutrition (TPN) 12 hours per day. About three months prior to diagnosis of RMS, the patient was started on the recently approved medication, teduglutide (rDNA origin) (Gattex^R^), which is indicated for the treatment of adult patients with SBS who are dependent on parenteral support [6–8]. Teduglutide is an analog of human glucagon-like peptide-2 (GLP-2) that is resistant to cleavage and inactivation by dipeptidyl peptidase IV, which increases intestinal and portal blood flow and induces intestinal growth. In a randomized, placebo-controlled phase III trial, a significantly greater proportion of patients with SBS receiving teduglutide were able to reduce weekly TPN volume requirements >20% and experienced gain in weight compared to patients treated with placebo; however, none of the patients was able to wean off TPN [7].

## 3. Treatment

**Figure 2:**
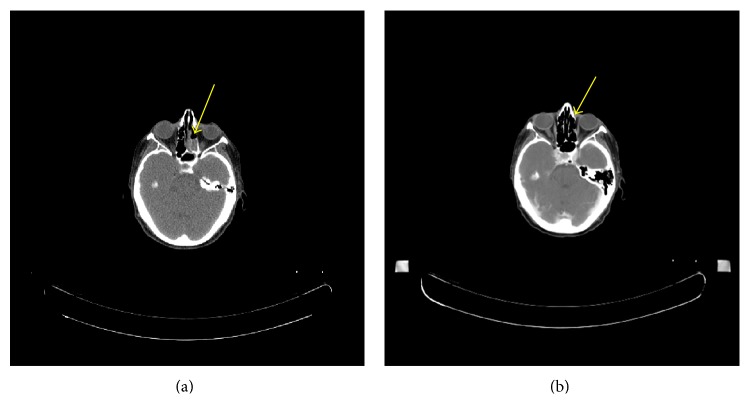
Images from computed tomography scanning prior to starting chemotherapy and after three cycles, showing resolution of left nasopharyngeal mass.

Once the diagnosis was established, teduglutide was discontinued due to the potential risk of acceleration of neoplastic growth. The patient began combination chemotherapy with doxorubicin, vincristine, and cyclophosphamide. Early cycles were complicated by neutropenic fever, oral stomatitis, and pancytopenia; all toxicities were managed with supportive measures and the patient completed a total of 9 months of therapy. Positron-emission tomography-CT scan after 3 cycles of chemotherapy showed dramatic improvement in multiple FDG-avid lesions, consistent with response to therapy. Rhinorrhea and nasal congestion resolved, and multiple masses including the left nasopharyngeal sarcoma regressed (Figure 2). After 9 months of chemotherapy, the patient had a clinical and radiographic complete response.

## 4. Discussion

The alveolar subtype of rhabdomyosarcoma is uncommon in adults and rarely occurs past the age of 65 years [9]. Chromosomal translocation involving PAX3 and FOXO1 is seen in about 70% of ARMS; a less frequent variant translocation (PAX7/FOX01) is present in 10–15% of ARMS [2, 10]. These reciprocal translocations fuse DNA-binding domains of PAX3 or PAX7 with the transactivation domain of FOX01, and the resultant novel products function as oncogenic proteins by promoting cell transformation and inhibiting apoptosis and myogenic differentiation. The fusion proteins and insulin-like growth factor-2 may cooperate in ARMS to promote a malignant phenotype [11]. The transmembrane receptor for insulin-like growth factor-1 (IGF-1) is frequently overexpressed in ARMS and is implicated in cell proliferation and metastatic behavior [12]. About 80% of ARMS express IGF-1 receptor, and* in vitro* studies suggest IGF-1 induces expression of myogenin, promotes cell proliferation, and prevents terminal muscle differentiation [13]. Inhibition of signaling through the IGF-1 receptor has been associated with significant reduction in ARMS growth* in vitro* [14, 15].

Teduglutide binds to GLP-2 receptors located in intestinal enteroendocrine cells, subepithelioid myofibroblasts, and enteric neurons of the submucosal and myenteric plexus [8]. Activation of these receptors causes local release of insulin-like growth factor- (IGF-) 1, nitric oxide, and keratinocyte growth factor (KGF) which promotes growth of intestinal epithelium [8]. GLP-2 indirectly stimulates intestinal growth through the IGF-1 pathway; both secretion of IGF-1 and expression of the IGF-1 receptor in intestinal epithelium are required for the proliferative effects of teduglutide on intestine [16, 17].

Common side effects of teduglutide occurring in more than 10% of patients include abdominal pain, abdominal distention, nausea, vomiting, change in gastrointestinal stoma, fluid overload, and upper respiratory tract infection [7]. The full prescribing information for teduglutide contains a warning about the potential for acceleration of neoplastic growth. In a 2-year carcinogenicity study in rats at subcutaneous doses of 3, 10, and 35 mg/kg/day (about 60, 200, and 700 times the recommended daily human dose of 0.05 mg/kg, resp.), teduglutide caused statistically significant increases in the incidences of adenomas in the bile duct and jejunum of male rats [8]. However, no dysplastic transformation was seen in intestinal tissue in patients treated in the phase III trial [18]. In a long-term follow-up study of teduglutide, 3 patients were diagnosed with cancer (adenocarcinoma of unknown primary, lung adenocarcinoma, and squamous cell lung cancer), but all three had predisposing risk factors for malignancy [18].

To our knowledge, we describe the first case of ARMS associated with the use of teduglutide. A causative role for the peptide in development of ARMS is unlikely because of the relatively short interval between initiation of teduglutide and diagnosis of sarcoma. Use of teduglutide may have contributed to growth of the malignancy, indirectly, through release of IGF-1, although a causative role for teduglutide in ARMS growth has not, to our knowledge, been established. As more biologic agents and mimetics become available to treat benign disease, physicians need to consider the potential roles of these agents in the pathogenesis of malignancy or acceleration of a previously existing or undiagnosed cancer. For example, a high density of GLP-2 receptors has been detected in the majority of gastrointestinal stromal tumors (GIST) in contrast to a wide range of cancers examined [19]. The biologic and pathologic implications of activation of GLP-2 in GIST need to be established to determine if teduglutide is contraindicated in patients with GIST. In summary, we present a case of metastatic ARMS occurring in a sexagenarian that underwent complete regression following cessation of teduglutide and treatment with sarcoma chemotherapy.
